# Proteomic Characterization of Cerebrospinal Fluid from Ataxia-Telangiectasia (A-T) Patients Using a LC/MS-Based Label-Free Protein Quantification Technology

**DOI:** 10.1155/2011/578903

**Published:** 2011-06-23

**Authors:** Monika Dzieciatkowska, Guihong Qi, Jinsam You, Kerry G. Bemis, Heather Sahm, Howard M. Lederman, Thomas O. Crawford, Lawrence M. Gelbert, Cynthia Rothblum-Oviatt, Mu Wang

**Affiliations:** ^1^Department Biochemistry and Molecular Biology, Indiana University School of Medicine, 635 Barnhill Dr., MS 4053, Indianapolis, IN 46202, USA; ^2^Division of Pediatric Allergy and Immunology, Departments of Pediatrics, Medicine, and Pathology, Johns Hopkins University School of Medicine, Baltimore, MD 21287, USA; ^3^Division of Pediatric Neurology, Departments of Neurology and Pediatrics, Johns Hopkins University School of Medicine, Baltimore, MD 21287, USA; ^4^Lilly Research Laboratories, Eli Lilly and Company, Indianapolis, IN 46285, USA; ^5^Research Department, A-T Children's Project, Coconut Creek, FL 33073, USA

## Abstract

Cerebrospinal fluid (CSF) has been used for biomarker discovery of neurodegenerative diseases in humans since biological changes in the brain can be seen in this biofluid. Inactivation of A-T-mutated protein (ATM), a multifunctional protein kinase, is responsible for A-T, yet biochemical studies have not succeeded in conclusively identifying the molecular mechanism(s) underlying the neurodegeneration seen in A-T patients or the proteins that can be used as biomarkers for neurologic assessment of A-T or as potential therapeutic targets. In this study, we applied a high-throughput LC/MS-based label-free protein quantification technology to quantitatively characterize the proteins in CSF samples in order to identify differentially expressed proteins that can serve as potential biomarker candidates for A-T. Among 204 identified CSF proteins with high peptide-identification confidence, thirteen showed significant protein expression changes. Bioinformatic analysis revealed that these 13 proteins are either involved in neurodegenerative disorders or cancer. Future molecular and functional characterization of these proteins would provide more insights into the potential therapeutic targets for the treatment of A-T and the biomarkers that can be used to monitor or predict A-T disease progression. Clinical validation studies are required before any of these proteins can be developed into clinically useful biomarkers.

## 1. Introduction

A-T is a neurodegenerative disease characterized by progressive cerebellar degeneration, immunodeficiency, cancer predisposition, premature aging, growth retardation, gonadal atrophy, high sensitivity to ionizing radiation, and genomic instability [1–6]. Many studies have suggested that a deficiency in the ability to repair DNA double-strand breaks (DSBs) is the main cause of the A-T phenotype [7]. A major breakthrough in understanding the pathophysiology of A-T came with the identification of the defective gene, ATM (ataxia-telangiectasia mutated), which is mutationally inactivated in individuals with the disease [8]. The identification of ATM has facilitated rapid progress in understanding many aspects of the molecular basis of this disease. 

The ATM protein is a serine-threonine kinase that undergoes autophosphorylation in response to DNA damage and subsequently initiates a signaling cascade that involves the phosphorylation of several down-stream substrates, including p53, p53BP1, Chk2, BRCA1, and TRF2 [7, 9, 10]. Recently, substantial insight has been obtained regarding the mechanism by which ATM signals to effector proteins after DNA double-strand breaks have occurred. Although ATM is an essential factor for sensing and signaling the repair of DSBs, other factors such as the MRN complex (Mre11/Rad50/Nbs1) may play an important upstream role in the activation of ATM [11]. In addition, ATM is a member of a large protein complex called the BRCA1-associated genome surveillance complex, suggesting that DNA damage recognition and signaling also involve other proteins, several of which are substrates for ATM [12]. A vast amount of literature has demonstrated the role of ATM in regulating a damage response pathway that ultimately leads to cell cycle checkpoint arrest, DNA repair, or apoptosis [13]. Understanding this role of ATM has explained the predisposition of A-T patients to develop immunodeficiency and cancer but has not explained the observed neurodegeneration. A global quantitative analysis of proteins associated with the A-T phenotype from A-T patient samples has not yet been reported but might shed new light on this dilemma.

One of the goals of proteomics is to measure and characterize the protein expression profiles in specific tissues and biofluids. Even though a tremendous effort has been made to improve proteomic technologies, there are still numerous challenges associated with even the most advanced technologies when analyzing global protein expression due to the inherent complexity of clinically relevant biological samples. These challenges include: (1) the sensitivity of the instrument and its ability to identify novel proteins, (2) the need to be moderate to high throughput, (3) the wide range of protein masses and abundances (dynamic range) that need to be covered, (4) the ability to quantitatively analyze protein expression and posttranslational modifications, and (5) access to the appropriate tissue and/or biofluid. With the recent development of a label-free protein quantification technology [14], large-scale and highthroughput analysis of complex biological samples has become possible which has overcome some of the challenges in proteomics [15–18]. This unique technology combines a proprietary sample preparation protocol [19], the LC/MS method, and statistical data analysis tools to quantitatively analyze proteins from whole tissue homogenates, cell lysates, or depleted serum/plasma samples. 

In this work, we used cerebrospinal fluid (CSF) samples from A-T patients and age- and gender-matched unaffected controls to identify and verify potential biomarkers of A-T. CSF was selected as it has been shown to be a relevant biological sample to study other neurodegenerative diseases such as Alzheimer's disease (AD), and to study changes in the predominant clinical phenotype of A-T (neurodegeneration) that have not been addressed in previous studies [20–22].

## 2. Materials and Methods

### 2.1. Reagents and Chemicals

Urea, ammonium carbonate, ammonium bicarbonate, mass-spectrometry grade formic acid, 2-iodoethanol, triethylphosphine and ProteoPrep spin cartridges were all purchased from Sigma-Aldrich (St. Louis, Mo, USA). Modified trypsin was purchased from Promega (Madison, Wis, USA). HPLC-grade water and acetonitrile were from Burdick & Jackson (Muskegon, Mich, USA).

### 2.2. Human Subjects

We contacted all adults (≥18 years of age) with A-T followed at the A-T Clinical Center at Johns Hopkins Medical Center. Eight were willing to have a lumbar puncture for the purpose of this research. The diagnosis of A-T was made by the combination of three observations: (1) characteristic neurologic abnormalities such as oculomotor apraxia, bulbar dysfunction and postural instability, (2) occulocutaneous telangiectasia, and (3) at least two of the following laboratory abnormalities: elevated serum alpha-fetoprotein level, absence of ATM on a western blot, increased rate of X-ray induced chromosomal breakage in comparison to a control population, and/or mutations in both alleles of the ATM gene.

Controls were otherwise healthy individuals having a lumbar puncture performed for a clinical indication (e.g., suspected pseudotumor cerebri or evaluation of chronic headache) and found not to have another neurologic disease.

The institutional review board of the Johns Hopkins Medical Institutions approved the study, and informed consent was obtained from every subject.

### 2.3. CSF Samples

The CSF samples were collected at Johns Hopkins Hospital (Baltimore, Md, USA). Lumbar punctures were performed by standard clinical technique using local anesthetic. The first 4 mL of CSF were used for standard chemistry and hematology tests. The next 1 mL was collected for proteomic analysis, immediately transported to the laboratory, and frozen at −80°C. 

The A-T group consisted of eight patients, six women and two men, ranging in age from 20 to 26 years old (mean ± S.D., 22.17 ± 2.13 years) (Table 1). As determined by the Bradford protein assay [23], the total CSF protein concentration in all samples ranged from 211.5 *μ*g/mL to 441.5 *μ*g/mL with a mean of 288.5 ± 93 *μ*g/mL. The control group consisted of five gender- and age-matched healthy controls. In the control group, the mean CSF protein concentration was 200.7 ± 81 *μ*g/mL, ranging from 98.9 *μ*g/mL to 369.9 *μ*g/mL. Aliquots of CSF were stored in polypropylene tubes at −80°C until use.

### 2.4. Sample Preparation

The two most abundant serum proteins, albumin, and IgG were removed from the CSF samples using ProteoPrep spin columns. Depleted CSF samples were denatured by 8 M urea for 1 h with agitation at room temperature. Chicken lysozyme (0.25 *μ*g, used as QA/QC reagent) and a volatile reduction/alkylation solution (97.5% acetonitrile, 2% iodoethanol, and 0.5% triethylphosphine) were added to each sample, and the solutions were incubated at 37°C for 1 h according to a previously published procedure [19]. The samples were dried under vacuum on a speed-vac. The resulting pellets were redissolved in 100 *μ*L of 100 mM ammonium bicarbonate (pH 8) containing 0.4 *μ*g of modified trypsin (Promega) and incubated for 4 h at 37°C. After a second addition of trypsin, the samples were incubated overnight at 37°C. All samples containing the tryptic peptides were filtered through a 0.45-*μ*m filter (Millipore, Billerica, Mass, USA) to avoid column clogging in LC/MS analysis.

### 2.5. LC-MS/MS Analysis

Tryptic peptides (2 *μ*g) were analyzed by a Thermo-Fisher Scientific LTQ linear ion-trap quadrupole mass spectrometer interfaced with a nano-electrospray ion source built in-house. The trapping column (NanoEase, C_18_ Column, 18 *μ*m × 23.5 mm, Waters, Milford, Mass, USA) and analytical column (nanoACQUITY UPLC BEH C_18_ Column, 1.7 *μ*m, 100 *μ*m × 100 mm, Waters) were used for peptide separation. Solvent A contained 99.9% HPLC-grade water and 0.1% formic acid, and solvent B contained 99.9% HPLC-grade acetonitrile and 0.1% of formic acid. The sample injection orders were randomized. The peptides were eluted with a linear gradient from 5 to 50% solvent B developed over 150 min at a flow rate of 200 nL/min, and effluent was electrosprayed into the LTQ mass spectrometer. The data were collected in “Triple-Play” mode (MS scan, Zoom scan, and MS/MS scan).

### 2.6. Protein Identification and Quantification

Protein database searches against the International Protein Index (IPI) human database (v3.60) and the NCBI Non-redundant-homo sapiens database (updated in June 2009) were carried out by both the SEQUEST (Thermo-Fisher Scientific, Waltham, Mass) and X!Tandem (an open-source software available from the Global Proteome Machine Organization, http://www.thegpm.org/) database searching algorithms. Identified proteins were categorized into two priority groups based on the quality of the peptide identification and the number of unique peptides identified [24]. All the proteins were identified with at least one best peptide identified at a confidence level ≥90% (*q*-value ≤ 0.1, *q*-value represents a false-discovery-rate or FDR which was described previously [14, 21]) or higher. Proteins were assigned to Priority 1 if two or more unique peptides were identified or Priority 2 if only a single peptide was identified. Peptides assigned to proteins with a confidence level of less than 90% were filtered out of this study. The estimation of the confidence levels, which is based on a random forest recursive partition supervised learning algorithm was described previously [24].

Protein quantification was carried out using a proprietary protein quantification algorithm licensed from Eli Lilly & Company (Indianapolis, Ind, USA) as described previously [14]. Briefly, once the raw files were acquired from the LTQ, all extracted ion chromatograms (XICs) were aligned by retention time. To be used in the protein quantification procedure, each aligned peak must match the parent ion, charge state, fragment ions (MS/MS data), and retention time (within a 1-min window). After alignment, the area-under-the-curve (AUC) for each individually aligned peak from each sample was measured, quantile normalized [25], and compared for relative abundance. All peak intensities were transformed to a log_2_ scale before quantile normalization. Quantile normalization was employed to ensure that every sample has a peptide intensity histogram of the same scale, location, and shape. This normalization removes trends introduced by technical variations including sample handling, sample preparation, total protein differences, and changes in instrument sensitivity while running multiple samples [25]. If multiple peptides have the same protein identification, then their quantile normalized log_2_ intensities were averaged to obtain log_2_ protein intensities. The log_2_ protein intensity is the final quantity that is fit by a separate ANOVA statistical model for each protein

(1) $$\begin{array}{c} {\text{Log}}_{2}\left(\text{Intensity}\right)=\;\text{Group}\;+\;\text{Sample}\left(\text{Group}\right). \end{array}$$

Sample(Group) is a random effect. Group effect refers to the effect caused by the experimental conditions or treatments being evaluated. Sample effect represents the random effects from individual biological samples. It also includes random effects from sample preparation. All of the injections were randomized, and the same person operated the instrument for all samples in this study. The inverse log_2_ of each sample's mean was calculated to determine the fold-change between groups.

### 2.7. Linear Discriminant Analysis (LDA)

LDA was performed using JMP (version 8) to separate the A-T group from the control group. The individual protein intensities of 13 Priority 1 proteins that showed significant expression changes were used as input for this analysis. The least number of proteins that gave the best discrimination between the two groups were selected as biomarker candidates.

### 2.8. Pathway Analysis

After LDA, a list of five proteins that could be used to distinguish A-T from normal samples was created and analyzed by Pathway Studio (v6.0) (Ariadne, Rockville, Md, USA) in an attempt to link them with the key A-T protein ATM. Briefly, the proteins' corresponding gene list was run against the ResNet database that is equipped with functional relationships from other scientific literature and commercial databases. The filters that we applied included “Add shortest path” and “Protein.” Protein interactions and the biological processes in which they were involved were noted. The information received was further explored in the literature to determine the interactions and regulatory relationships between the proteins of interest and ATM.

### 2.9. Multiple-Reaction-Monitoring (MRM) Analysis

To verify and validate the candidate biomarkers of A-T, an MRM-based targeted proteomic analysis was performed to quantify the relative protein expression levels between the control and A-T patient samples. An AB/SCIEX 4000 QTRAP mass spectrometer interfaced with a Dionex Ultimate 3000 HPLC system was used for this targeted proteomic quantification study. In this study, five candidate proteins (listed in Table 4) were selected for verification/validation. The analytes, which were the same tryptic peptides used for the label-free discovery study, were first loaded onto a trapping column (75 *μ*m i.d. × 20 mm) and then onto an analytical column (75 *μ*m i.d. × 150 mm packed in-house with C_18_ 3 *μ*m reversed phase resin), where they were eluted using a gradient of 5–45% acetonitrile with 0.1% formic acid at a flow rate of 300 nL/min over 60 min. Source temperature was set at 160°C, and source voltage was set at 2400 V. The collision energy (CE) for each transition was calculated from the equations CE = 0.05* (m/z) + 8 for (M + 2H^+^) ions and CE = 0.044* (m/z) + 8 for (M + 3H^+^) ions. The declustering potential (DP) was set at 100 V, and a dwell time of 20–30 ms was used to maximize the number of transitions per MRM experiment. All MRM peptides and transitions are shown in Table 4. Relative quantification was accomplished using the Analyst software (version 1.5.1 Applied Biosystems).

## 3. Results

To characterize the alterations in protein expression related to A-T, we performed LC/MS-based quantitative proteomic analysis of CSF from control and A-T patients. The patient information in each group is summarized in Table 1. Proteins identified based on priority groups are summarized in Table 2. A total of 477 proteins were identified and quantified with high confidence in the samples. The expression levels of 13 proteins from Priority 1 and 7 proteins from Priority 2 were statistically significantly changed (listed in Table 3). The 13 significantly changed proteins from the Priority 1 group were further analyzed by Linear Discriminant Analysis (LDA) and pathway analysis for their roles in biological processes.

**Figure 1:**
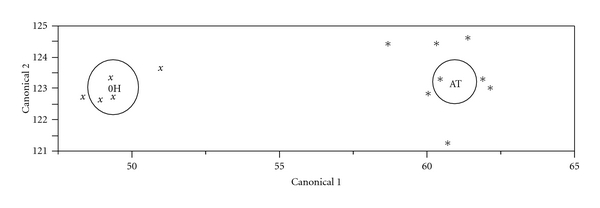
Linear Discriminant Analysis (LDA) of differentially expressed proteins in A-T. A panel of 13 differentially expressed proteins were analyzed by LDA (a function of JMP version 8 software). Expression differences of proenkephalin-A (P01210), isoform 1 of extracellular matrix protein 1 (Q16610), secretogranin-2 (P13521), isoform 1 of CD166 antigen (Q13740), and insulin-like growth factor binding protein 7 (Q16270) can clearly discriminate A-T samples (AT, ∗, 8 samples) from normal controls (0H, *x*, 5 samples), suggesting that these five proteins can potentially serve as a panel of biomarkers of A-T.

**Figure 2:**
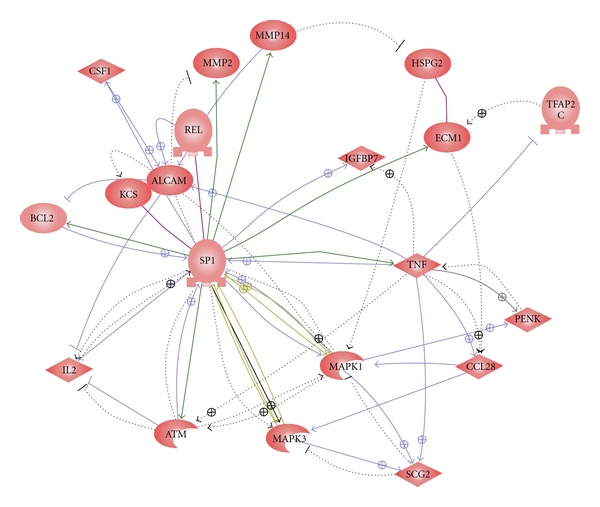
Pathway analysis linking five A-T biomarker candidates and ATM. Pathway studio was used to link the A-T biomarker candidates with ATM. Direct interactions are represented by solid lines, whereas indirect interactions are shown in dashed lines.

**Figure 3:**
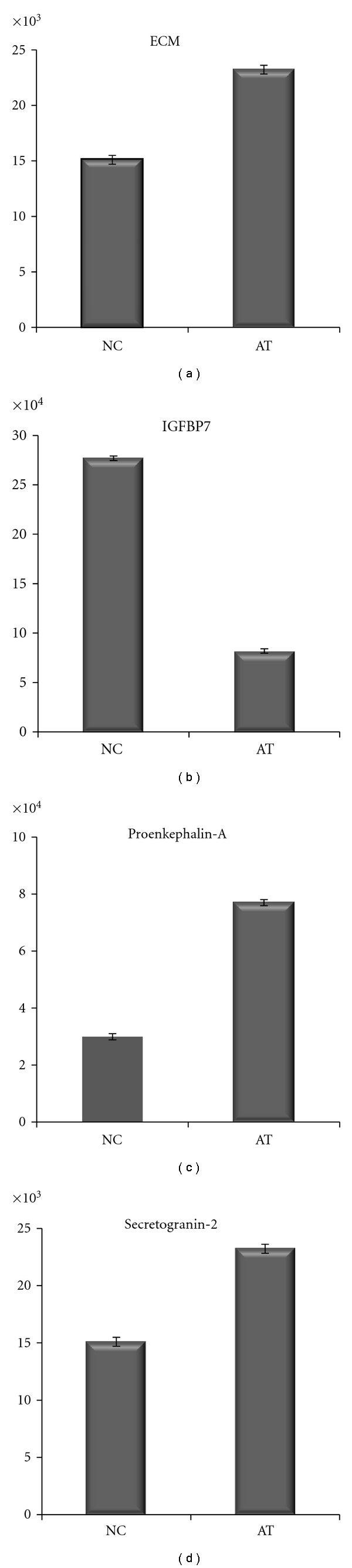
MRM analysis of A-T biomarker candidates. Relative protein expression levels were determined by averaging the area-under-the-Curve (AUC) for each selected MRM transition for each peptide shown in Table 4. For secretogranin-2 and IGFBP7, the fold-changes from both MRM peptides were also averaged. Statistical analysis was performed by ANOVA models using PROC MIXED in SAS. *P* < .05.

Figure 1 illustrates the LDA results. Expression differences of proenkephalin-A (PENK, P01210), isoform 1 of extracellular matrix protein 1 (ECM1, Q16610), secretogranin-2 (SCG2, P13521), isoform 1 of CD166 antigen (ALCAM, Q13740), and insulin-like growth factor binding protein 7 (IGFBP7, Q16270) can clearly discriminate A-T samples from healthy controls.The literature search results demonstrate that these five proteins are involved in either human cancers or neurodegenerative processes [26–42].  Figure 2 shows a protein-protein interaction network linking these five proteins to ATM from the pathway analysis. Their relative protein expression levels as determined by MRM are shown in Figure 3.

For QA/QC purpose, chicken lysozyme was spiked into every individual sample at a constant amount (10 ng chicken lysozyme/2 *μ*g of sample) before tryptic digestion. There were 9 unique chicken lysozyme peptides being detected and quantified. After averaging these peptide concentration values, a −1.099 fold-change was observed with a *q*-value of 0.77 (77% FDR), suggesting this observed small change is not statistically significant and the data obtained from this study was reliable.

MRM results demonstrate the same direction of protein expression changes (up- or downregulation) as compared to the global discovery study, even though the absolute fold-change may be slightly different in some cases, likely due to differences in the platform used. In this targeted proteomic study, we were able to detect and quantify four out of the five proteins of interest. Unfortunately, we were unable to confidently detect the MRM peptide “SSPSFSSLHYQDAGNYVCETA” from ALCAM due to its low abundance. All of the MRM peptides and transitions for each protein of interest are listed in Table 4.

## 4. Discussion

Much of the effort in proteomics has been devoted to improve the sensitivity of the instrument and measurement accuracy. At the present time, there is no consensus within the field of proteomics on any one technology that can attain complete and quantitative protein coverage of all proteins in a given tissue or biofluid. The most commonly used proteomic approach, the so called “bottom-up” approach, utilizes a two-step approach: peptide separation followed by peptide/protein identification and quantification by mass spectrometry (MS). Two-dimensional gel electrophoresis (2DE) has been the workhorse for protein separation in proteomics research efforts in the past decade, but its inability to widen the protein dynamic range and its low throughput remain its biggest disadvantages and thus limit its utility in large-scale and highthroughput proteomic analysis. 

One alternative approach to 2DE is the nongel-based liquid chromatography mass spectrometry-based shotgun proteomic technology [43–46]. It provides a powerful analytical platform to resolve and identify thousands of proteins from a complex biological sample in a single experiment. This approach is rapid and more sensitive, and it increases the protein dynamic range 3- to 4-fold as compared to 2DE. The hallmark of this method is its ability for high-throughput large-scale proteomic analysis [47, 48]. Although some success using isotopic labeling technology in combination with mass spectrometry for protein quantification has been reported [48], recently developed label-free protein quantification technology [14] has become a major platform for biomarker discovery primarily due to the high costs of the labeling reagents, especially for a large-scale study. In this study, we used a peak-intensity-based label-free protein quantification method that was previously applied for many other studies [14, 15, 17, 18].

One challenge to studying the neurodegeneration seen in A-T is access to affected brain tissue. For this reason, we chose CSF to analyze since this biofluid is in direct contact with the brain and studies of other neurodegenerative diseases have shown that disease-specific changes in the brain can be detected [20–22]. A recent study by Cheema et al. [49], using analysis of ATM-mediated gene and protein expression in A-T fibroblasts found a completely different set of proteins than those observed in our CSF study and highlights the importance of selecting a clinically relevant tissue for biomarker discovery.

### 4.1. Confidence in the Methodology

In this proteomic study, we did not detect A-T-related proteins, such as ATM-related protein kinases or their substrates [7]. This could be due to the inability of current LC/MS technology to confidently detect low-abundance proteins. However, the advantages of the method far outweigh this limitation. Firstly, proteomic analysis ignores transcripts that may never be translated by detecting only the end products of gene activity, giving it an advantage over genomic analysis. Secondly, the LC/MS-based label-free protein quantification technology used here has proven itself a powerful tool to resolve and identify thousands of proteins from complex biological samples [16, 50]. It is a method that compares the relative expression levels of the same protein under different physiological conditions. The method is rapid highthroughput, and more sensitive than many other proteomic platforms [16], and it increases the protein dynamic range 3- to 4-fold compared to the conventional 2D gel-based proteomic platform. During the development of the method, chicken lysozyme was used for QA/QC purposes, and the method has since been robustly tested on many different types of samples [15–18]. Automation allows it to be applied to large-scale proteomic analysis; thus, it has become a tool of choice for biomarker discovery [15, 51]. The inclusion of statistics in both the experimental design and data analysis allows for the detection of small but statistically significant changes not offered by other methods. We are, therefore, confident in the qualitative and quantitative data produced by this method.

### 4.2. Significance of Results

**A. Statistical Motivation**

The size of the treatment or disease effect (signal) needs to be evaluated relative to the sample and replicate variation (noise). The signal to noise ratio is estimated based on a statistical model. If the data have multiple sources of random variation such as biological samples and replicates then the data are modeled as a Linear Mixed Model (A generalization of an ANOVA, Analysis of Variance). This kind of model, especially when applied to complex experimental designs, cannot be handled by introductory methods such as *t*-tests. The exact scale of the protein expression used in the model can make a difference in the sensitivity. There is usually a large technical variation introduced by the act of “measurement” in any “omics” study. Randomization of measurement order will eliminate the bias, but it is still extremely important to “normalize” or mathematically calibrate the measurement. This is a highly technical matter but can be viewed as similar to mathematically resetting a scale to zero before each measurement. We use a statistically based method called “quantile normalization” [25] which was the result of considerable research on genomic data. Because “omics” measures of expression are usually on an arbitrary scale, it is best to evaluate ratios or their equivalent differences on the log scale. Log base 2 is chosen because a unit difference on the log scale is equivalent to a two-fold change.

**B. Five Biomarker Candidate Proteins**

From the LDA, five candidate proteins whose relative expression levels could be used to precisely discriminate control samples from A-T patient samples were discovered. After reviewing the literature, all of these proteins were found to play some role in either cancer or neurodegenerative processes, or both, which lends support to these proteins being viable biomarkers of A-T.

The first of these five proteins is proenkephalin-A (PENK), which is an opioid neuropeptide precursor, a neuroendocrine hormone, and a cytokine. It is involved in pain perception, modulation of the immune system, anticonvulsant activity, and the neurodegenerative disorder Huntington's disease [27, 30]. It is also involved in several cancers, including breast cancer and prolactin-secreting pituitary adenoma [26, 28, 29]. This protein was found 30% overexpressed in A-T samples.

Isoform 1 of Extracellular matrix protein 1 (ECM1), which is 42% over-expressed in A-T samples, is involved in many cancers, including breast, esophageal, laryngeal, thyroid, and lung cancers and may play a role in angiogenesis [31]. It is mutated in lipoid proteinosis, a dermatological disease in which patients may develop neurological abnormalities such as temporal lobe epilepsy and mental retardation [32].

The third protein, the neuroendocrine prohormone secretogranin 2 (SCG2), has a role in both neurological processes and cancer. SCG2 is over-expressed by 35% in the A-T patients and is involved with the packaging and sorting of peptide hormones and neuropeptides into secretory vesicles. One of its gene products promotes neuroprotection and neuronal plasticity in mice and humans [38]. Secretogranin 2 has also been suggested to be involved in neuroendocrine tumors and amyotrophic lateral sclerosis (a neurodegenerative disorder) [39, 40].

The fourth protein, isoform 1 of CD166 antigen (ALCAM), which has a role in cancer and neurological disorders [33–37], was found to be 52% over-expressed in the A-T samples. It is involved in neurite extension by neurons in chickens [35] and in the neurodegenerative disorder multiple sclerosis [34]. In addition, CD166 plays a role in many cancers, including melanoma, prostate, colorectal, pancreas, and breast [33, 36, 37].

The final protein, insulin-like growth factor binding protein 7 (IGFBP7), is downregulated 46% in A-T compared to control samples. It plays a role in regulating proliferation, differentiation, and apoptosis. Additionally, it is involved in several types of cancers, including colorectal and inflammatory breast cancers [41, 42].

**C. Other Priority 1 Proteins**

The remaining eight significantly changed proteins in the Priority group 1 are SPARC (secreted protein acidic and rich in cysteine), neurosecretory protein VGF, TPP1 (cDNA FLJ56402, highly similar to tripeptidyl-peptidase 1), neurocan core protein, chromogranin A, cathepsin D, SOD3 (extracellular superoxide dismutase), and ENPP2 (isoform 1 of ectonucleotide pyrophosphatase/phosphodiesterase family member 2) (Table 3). Among these proteins, SPARC, neurosecretory protein VGF, TPP1, and SOD3 are of particular interest because they have been implicated in neurodegenerative processes.

SPARC is a unique matricellular glycoprotein involved in many types of diseases including cancer, inflammation, and neurodegeneration [52–54]. Its function is associated with cell development, remodeling, cell turnover, and tissue repair. Our observed downregulation (1.47-fold) of this protein in A-T patients implicates deficiencies associated with these cellular functions in this disease.

Neurosecretory protein VGF has been identified by many proteomic studies [55–57]. It plays a role in neuronal communication [56]. This gene is specifically expressed in a subpopulation of neuroendocrine cells and is upregulated by nerve growth factor [57]. However, its exact function remains to be discovered.

TPP1 (cDNA FLJ56402, highly similar to tripeptidyl-peptidase 1), also known as CLN2, is a member of the family of serine-carboxyl proteinases and plays a crucial role in lysosomal protein degradation; a deficiency in this enzyme leads to fatal neurodegenerative disease [58]. It is also involved in telomere protection [59]. Based on its known functions, TPP1 is expected to be down-regulated in A-T patients, which is what we observed (down-regulated 1.44-fold).

SOD3 is an antioxidant enzyme associated with many pathways and diseases. Its association with neurodegeneration has been reported previously in a study of antioxidant gene therapy [60]. SOD3's function is to protect against neurodegeneration. Down-regulation (1.37-fold) of this protein in A-T patients would suggest less of a protective effect by SOD3. Importantly, a large body of evidence suggests that oxidative stress plays some role in the pathophysiology of A-T. As a recent example, one group has shown that ATM can be directly activated by oxidation in the absence of DNA double-strand breaks, implying that ATM may act as a redox sensor capable of regulating cellular responses to oxidative stress as well as genotoxic stress [61].

## 5. Conclusion

We identified novel CSF biomarker candidates for A-T from the 13 priority 1 proteins with significant absolute fold-changes of at least 1.3 (30% increase or decrease) (*q* < 0.05). LDA was applied to assess the ability of individual and/or combinations of these proteins to correctly classify individuals into the control or disease group. The selectivity and specificity from the LDA was high, suggesting that it is possible to correctly assign individuals to the proper group (control or A-T patient) when the expression levels of these biomarker candidates are accurately measured in the CSF. Findings from our study confirm that the mass spectrometry-based label-free protein quantification and MRM technologies can be used successfully for biomarker discovery and validation. However, limitations of our study require us to interpret the data with caution. First, the current study constituted a small sample size, and further validation studies with a larger set of patient cohort samples are necessary. Second, the fold-changes observed in the study are relatively small, which require high measurement precision to produce high quality, clinically valid data. Thus, mass spectrometry-based methods may not be a practical approach for clinical applications. Third, CSF may not be an ideal biospecimens for prognostic applications due to the invasiveness involved in sample collection. Future studies involving serum or plasma samples would make this biomarker discovery strategy even more attractive with the hope that such noninvasive biospecimens can be incorporated into routine clinical practice and utilized in clinical trials for the assessment of potential therapeutic compounds.

## Figures and Tables

**Table 1:** Summary of the CSF samples used for the study.

Sample	Age	Gender	Type
Control 1	23	M	Pseudotumor cerebri
Control 2	28	M	Headaches
Control 3	32	F	Pseudotumor cerebri
Control 4	39	F	Pseudotumor cerebri
Control 5	44	F	Pseudotumor cerebri
A-T 1	21	F	A-T patient
A-T 2	21	F	A-T patient
A-T 3	26	F	A-T patient
A-T 4	20	M	A-T patient
A-T 5	22	M	A-T patient
A-T 6	23	F	A-T patient
A-T 7	20	F	A-T patient
A-T 8	22	F	A-T patient

**Table 2:** Overall summary of the proteomic analysis.

Protein priority	Peptide ID confidence	Multiple sequences	Number of proteins	Number of significant changes	Maximum absolute fold-change	Median %CV (replicate)^(a)^	Median %CV (rep + sample)^(b)^
1	High (>90%)	Yes	204	13	1.52	11.97	19.16
2	High (>90%)	No	273	7	4.30	36.60	47.06
Overall			477	20	4.30	24.29	33.11

^(a)^Replicate %CV represents technical variations.

^(b)^Rep + sample %CV represents overall variations including both technical and biological variations.

**Table 3:** Priority 1 and Priority 2 proteins.

Protein name	Priority group	Swiss-Prot accession number	Peptides used for protein identification	Peptide ID confidence (%)	Fold-Change (negative values indicate downregulation)^(a)^
			LVRPADINFLACVMECEGKLPSLK	>99.99	
			AEEDDSLANSSDLLKELLETGDNR	>99.99	
Proenkephalin-A	1	P01210	ECSQDCATCSYR	93.45	1.29
			ELLETGDNR	99.31	
			ELLQLSKPELPQDGTSTLR	99.23	

			FSCFQEEAPQPHYQLR	>99.99	
Isoform 1 of Extracellular matrix protein 1	1	Q16610	QHVVYGPWNLPQSSYSHLTR	>99.99	1.42
			NIWRDPALCCYLSPGDEQVNCFNINYLR	>99.99	
			ACPSHQPDISSGLELPFPPGVPTLDNIK	>99.99	

			IESQTQEEVR	>99.99	
			TNEIVEEQYTPQSLATLESVFQELGK	>99.99	
			VLEYLNQEK	>99.99	
Secretogranin 2	1	P13521	GQGSSEDDLQEEEQIEQAIK	>99.99	1.35
			EHLNQGSSQETDKLAPVS	>99.99	
			ALEYIENLR	90.56	
			QYWDEDLLMK	99.73	

Isoform 1 of CD166 antigen	1	Q13740	SSPSFSSLHYQDAGNYVCETALQEVEGLK	>99.99	1.52
			ALFLETEQLK	91.12	

			ITVVDALHEIPVK	>99.99	
			SSSDTCGPCEPASCPPLPPLGCLLGETR	>99.99	
			SRYPVCGSDGTTYPSGCQLR	>99.99	
Insulin-like growth factor binding protein 7	1	Q16270	TELLPGDRDNLAIQTR	99.98	−1.46
			EDAGEYECHASNSQGQASASAK	99.99	
			AGAAAGGPGVSGVCVCK	>99.99	
			GTCEQGPSIVTPPK	99.50	

			YIPPCLDSELTEFPLR	>99.99	
SPARC	1	P09486	APLIPMEHCTTR	99.96	−1.47
			PPCLDSELTEFPLR	99.52	
			RLEAGDHPVELLAR	>99.99	

			GRPEAQPPPLSSEHKEPVAGDAVPGPK	>99.99	
			LADLASDLLLQYLLQGGAR	>99.99	
			ESAREEEEAEQER	>99.99	
			NSEPQDEGELFQGVDPR	>99.99	
			RPESALLGGSEAGER	>99.99	
Neurosecretory protein VGF	1	O15240	THLGEALAPLSK	>99.99	1.42
			VGEEDEEAAEAEAEAEEAER	>99.99	
			LLQQGLAQVEAGR	>99.99	
			NAPPEPVPPPR	>99.99	
			GLQEAAEER	95.27	
			FGEGVSSPK	>99.99	
			AYQGVAAPFPK	>99.99	

TPP1	1	B5MDC1	YLTLENVADLVRPSPLTLHTVQK	98.74	−1.44
			VPIPWVSGTSASTPVFGGILSLINEHR	>99.99	

			GIEDEQDLVPLEVTGVVFHYR	>99.99	
Neurocan core protein	1	O14594	RNPQELYDVYCFAR	>99.99	1.30
			ELGGEVFYVGPAR	>99.99	

			HSGFEDELSEVLENQSSQAELK	>99.99	
			AEGNNQAPGEEEEEEEEATNTHPPASLPSQK	>99.99	
			GEQEHSQQKEEEEEMAVVPQGLFR	99.07	
			ELQDLALQGAK	>99.99	
			PQALPEPMQESK	>99.99	
Chromogranin A	1	P10645	SEALAVDGAGKPGAEEAQDPEGK	>99.99	1.31
			RPEDQELESLSAIEAELEK	>99.99	
			EDSLEAGLPLQVR	>99.99	
			YPGPQAEGDSEGLSQGLVDREK	>99.99	
			SGELEQEEER	>99.99	
			TDGARPQALPEPMQESK	>99.99	
			GLSAEPGWQAK	>99.99	

			AIGAVPLIQGEYMIPCEK	>99.99	
			ISVNNVLPVFDNLMQQK	>99.99	
			FDGILGMAYPR	>99.99	
Cathepsin D	1	P07339	VSTLPAITLK	98.91	−1.37
			TMSEVGGSVEDLIAK	>99.99	
			VGFAEAAR	90.97	
			LLDIACWIHHK	99.98	

			VTEIWQEVMQR	>99.99	
			LACCVVGVCGPGLWER	>99.99	
SOD3	1	P08294	AGLAASLAGPHSIVGR	96.66	−1.37
			AVVVHAGEDDLGR	>99.99	
			RDDDGALHAACQVQPSATLDAAQPR	>99.99	
			GGNQASVENGNAGR	>99.99	

			NGVNVISGPIFDYDYDGLHDTEDKIK	99.71	
			YDAFLVTNMVPMYPAFK	>99.99	
			SYTSCCHDFDELCLK	>99.99	
			NKLDELNKR	>99.99	
			RLHYANNR	>99.99	
			VNSMQTVFVGYGPTFK	>99.99	
			DIEHLTSLDFFR	>99.99	
ENPP2	1	Q13822	EIDKIVGQLMDGLK	>99.99	−1.36
			TEFLSNYLTNVDDITLVPGTLGR	>99.99	
			RWHVAR	92.27	
			SCGTHSPYMRPVYPTK	>99.99	
			SYPEILTLK	94.45	
			QAEVSSVPDHLTSCVRPDVR	99.79	
			GESHWVDDDCEEIK	>99.99	
			VMPNIEK	94.62	
			IEDIHLLVER	>99.99	
			VSPSFSQNCLAYK	99.85	
			KPDQHFKPYLK	99.79	
			PAVLYR	91.70	
			PAGFVRPPLIIFSVDGFR	>99.99	
			CFFQGDHGFDNK	90.16	
			QMSYGFLFPPYLSSSPEAK	99.03	

Insulin-like growth factor binding protein complex acid abile chain	2	P35858	LAYLQPALFSGLAELR	>99.99	−1.45

Caspase recruitment domain-containing protein 14	2	Q9BXL6	GALPGAK	95.91	−1.95

Endothelial cell-selective adhesion molecule	2	Q96AP7	QLPSFQTFFAPALDVIR	99.94	1.95

Isoform 1 of phosphatidylinositol 3-kinase regulatory subunit gamma	2	Q92569	LGEIHDSK	90.36	−2.16

Neutral amino acid transporter B	2	Q15758	SCTVLNVEGDALGAGLLQNYVDR	90.14	−1.87

Isoform 1 of latrophilin-1	2	O94910	TDDKICDADPFQMENVQCYLPDAFK	>99.99	1.57

Isoform 1 of histone-lysine N-methyltransferase MLL3	2	Q8NEZ4	IQPPIAQLPIK	91.36	2.03

^(a)^The fold-changes are the average values from all the peptides used for protein quantification; individual values from individual peptide are given in the supplementary data.

**Table 4:** MRM transitions for biomarker candidates.

Protein name	Swiss-Prot accession number	Peptides used for MRM validation assay	MRM transitions	Observed fold-change
			1063.7 (M + 2H^+^) → 634.4 (y6)	
Proenkephalin-A	P01210	ELLQLSKPELPQDGTSTLR	1063.7 (M + 2H^+^) → 974.5 (y9)	2.57
			1063.7 (M + 2H^+^) → 1313.7 (y12)	

Isoform 1 of Extracellular matrix protein 1 (ECM)			790.9 (M + 3H^+^) → 588.3 (y10)	
	Q16610	QHVVYGPWNLPQSSYSHLTR	790.9 (M + 3H^+^) → 871.9 (y15)	1.19
			790.9 (M + 3H^+^) → 953.5 (y16)	

			610.1 (M + 2H^+^) → 488.7 (y8^2+^)	
Secretogranin 2	P13521	IESQTQEEVR	610.1 (M + 2H^+^) → 976.5 (y8)	1.54
		VLEYLNQEK	568.6 (M + 2H^+^) → 794.4 (y6)	
			568.6 (M + 2H^+^) → 923.4 (y7)	

			478.9 (M + 3H^+^) → 560.8 (y10^2+^)	
		ITVVDALHEIPVK	478.9 (M + 3H^+^) → 610.4 (y11^2+^)	−3.38
			478.9 (M + 3H^+^) → 660.9 (y12^2+^)	
Insulin-like growth factor binding protein 7 (IGFBP7)	Q16270	AGAAAGGPGVSGVCVCK	746.9 (M + 2H^+^) → 783.4 (y7)	
			746.9 (M + 2H^+^) → 1036.5 (y10)	
			746.9 (M + 2H^+^) → 1150.6 (y12)	

Isoform 1 of CD166 antigen	Q13740	SSPSFSSLHYQDAGNYVCETA	Failed to detect this peptide	N/A
